# Polyphenol Composition of Extracts of the Fruits of *Laserpitium Krapffii* Crantz and Their Antioxidant and Cytotoxic Activity

**DOI:** 10.3390/antiox8090363

**Published:** 2019-09-01

**Authors:** Anna Bogucka-Kocka, Natalia Vorobets, Małgorzata Chrząszcz, Wioleta Pietrzak, Katarzyna Szewczyk

**Affiliations:** 1 Department of Biology and Genetics, Medical University of Lublin, 4a Chodźki Str., 20-093 Lublin, Poland; 2 Department of Pharmacognosy and Botany, Faculty of Pharmacy, Danylo Halytsky Lviv National Medical University, 69 Pekarska Str., Lviv 79010, Ukraine; 3 Department of Pharmaceutical Botany, Medical University of Lublin, 1 Chodźki Str., 20-093 Lublin, Poland

**Keywords:** *Laserpitium*, antioxidants, cytotoxic activity, LC-MS/MS, phenolics

## Abstract

During inflammation, reactive oxygen species (ROS) are produced in large amounts, causing oxidative stress. Several studies confirm that plant extracts rich in phenolic compounds may inhibit ROS production. For that reason, the aim of this work is the qualitative and quantitative analysis of phenolic acids and flavonoids in the etheric (LAEN) and methanolic (LAM) extracts of the fruits of *Laserpitium krapffii* Crantz, as well as their antioxidative and cytotoxic properties. Liquid chromatography–electrospray tandem mass spectroscopy (LC-ESI-MS/MS) enabled the identification of 12 phenolic acids and nine flavonoids. Both tested extracts scavenged ROS in a concentration-dependent manner. Stronger activity was observed for the methanolic extract. The cytotoxic effect of both extracts in increasing concentrations on five types of cancer cell lines was also investigated. The cytotoxicity was estimated using trypan blue vital staining. It was found that the analyzed extracts induced the apoptosis of the cells of all the tested cell lines. In conclusion, our results present that the fruits of *L. krapffii* can be a source of valuable compounds with protective effects against oxidative damage.

## 1. Introduction

*Laserpitium krapffii* Crantz (syn. *L. alpinum* Waldst. and Kit.) is a perennial herbaceous plant that is commonly known as Krapf’s laserwort. The species, belonging to the *Apiaceae* family, is widely spread throughout the northern regions of the Balkan Peninsula, and the southern and eastern parts of the Alps [1].

Reports regarding the chemical composition and biological activities of *L. krapffii* are limited. Primarily data indicated the content of only sesquiterpene lactones [2,3] and essential oils [4] in this plant.

The main group of secondary metabolites occurring in the *Laserpitium* L. genus is sesquiterpene lactones, which are mainly of the slovanolide-type class of guaianolides [3,5]. The exception is *L. krapffii*; these compounds are also isolated from the herb, fruits, and underground parts of *L. siler* [3,6], the rhizomes and roots of *L. orchidanum* and *L. zerney* [5], *L. archangelica* [7], and *L. prutenicum* [8]. The polyphenols isolated from the ethanolic extract of *L. latifolium* leaves included astragalin, avicularin, isoquercitrin, quercitrin, rutin, chlorogenic, and neochlorogenic acid; other unidentified quercetin, isorhamnetin, and kaempferol derivatives were also isolated [9]. Chemical studies of various *Laserpitium*s pecies have also shown the presence of daucane derivatives [10,11], phenylpropanoid derivatives [10], and essential oils [4,12,13,14].

Some *Laserpitium* species have been used in traditional European medicine as diuretics for treating liver and heart dysfunctions, gastrointestinal disorders, pulmonary tuberculosis, and rheumatism [9,15]. It has been reported that the extracts and isolated compounds from *L. zernyi* Hayek and *L. ochridanum* Micevski have cytotoxic activities on two human breast cancer cell lines [5]. Moreover, trilobolide and its acetoxy analog obtained from *L. siler* L. and *L. archangelica* Wulf. in Jacq. are strong activators of cytokine secretion [16], and daucane esters isolated from *L. latifolium* have anti-inflammatory potential [11]. Tirillini et al. [17] noticed that the oil from *L. garganicum* has a strong antifungal activity. The essential oil from the underground parts of *L. zernyi* and *L. ochridanum* have a significant antinociceptive effect and reduced paw edema [18].

Due to the biological importance of *Laserpitium* species and the negligible current knowledge about the chemical components and biological activity among these plants, the aim of the present study was to evaluate the cytotoxic and antioxidant properties of the extracts of the fruits of *L. krapffii*. Furthermore, the phenolic acids and flavonoids content in the investigated samples was also determined.

## 2. Materials and Methods

### 2.1. Plant Material

The fruits of *Laserpitium krapffii* Crantz were gathered at the top of the Sheshul Montenegro mountain ridge in the Rakhiv region of Zakarpatska oblast’ (Transcarpathian region) (Ukraine). The plant material was dried in the air, in shade, and at an average temperature 26.0 ± 0.5 °C [19].

### 2.2. Chemicals and Reagents

LC grade acetonitrile, ascorbic acid, 2,2-diphenyl-1-picrylhydrazyl radical (DPPH^•^), 2,2′-azino-bis-(3-ethyl-benzthiazoline-6-sulfonic acid) (ABTS^•+^), Folin–Ciocalteu reagent, nitrobluetetrazolium (NBT), xanthine, xanthine oxidase, and 5,5-dithiobis-(2-nitrobenzoic acid) (DTNB) were obtained from Sigma-Aldrich Fine Chemicals (St. Louis, MO, USA). Phosphate-buffered saline (PBS) was obtained from Gibco (Carlsbad, CA, USA). Reference compounds of phenolic acids and flavonoids were purchased from ChromaDex (Irvine, CA, USA). Water and formic acid for LC analysis were from Merck (Darmstadt, Germany). Sodium hypochlorite (NaOCl), ethylenediaminetetraacetic acid (EDTA), and all the other chemicals were of analytical grade and were purchased from the Polish Chemical Reagent Company (POCH, Gliwice, Poland).

### 2.3. Extraction Method

First, 50.0 g of dried and powdered fruits were extracted successively in the filter paper with petroleum ether (bp 45–60 °C; 300 mL) for over 40 h in a Soxhlet apparatus. Etheric extract was filtered and evaporated under reduced pressure, and then subjected to lyophilization using a vacuum concentrator (Free Zone 1 apparatus; Labconco, Kansas City, KS, USA) affording 4.2 g of dried fraction (LAEN). In the next stage, dried fruits (after petroleum ether extraction) were sonicated with a mixture of methanol–acetone–water (3:1:1, *v*/*v*/*v*) (3 × 100 mL) at a controlled temperature (40 ± 2 °C) for 45 min. Combined methanolic extracts were filtered, concentrated under reduced pressure, and lyophilized in a vacuum concentrator to obtain 11.3 g of dried residue (LAM). 

### 2.4. Solid Phase Extraction (SPE)

Both crude extracts (LAEN, LAM) were purified using an Solid Phase Extraction (SPE) system (Baker spe-12G™, J.T. Baker, Germany) and previously conditioned Bakerbond C-18 column (Octadecyl, 500 mg, J.T. Baker, Phillipsburg, USA). Then, 5 mL of extracts in 30% methanolic were eluted with 5 mL of water, followed by 5 mL of 50% and finally 5 mL of 80% methanol. The obtained extracts were evaporated to dryness and used for LC-MS/MS analysis.

### 2.5. Total Phenolic, Flavonoid, and Phenolic Acids Content

The total phenolic (TPC) and total flavonoid content (TFC) were determined using the colorimetric methods previously described [20]. The absorbance was measured at 680 nm and 430 nm, respectively, using an ELISA Reader. The total phenolic concentration was calculated from the calibrated curve (*R*^2^ = 0.9977), using gallic acid in a concentration of 0.001 to 0.008 mg/mL as a standard. The results were expressed as mg of gallic acid equivalent (GAE) per 1 g of dry extract (DE). TFC was calculated from the calibrated curve (*R*^2^ = 0.9999), using quercetin in a concentration of 0.05 to 0.20 mg/mL as a standard. The results were expressed as mg of quercetin equivalent (QE) per 1 g of DE. The total phenolic acids content (TPAC) was performed using the method with Arnov’s reagent, as described in Polish Pharmacopoeia IX [19]. The absorbance was measured at 490 nm. The total phenolic acids content was calculated from the calibrated curve (*R*^2^ = 0.9964), using caffeic acid in a concentration of 3.36 to 23.52 μg/mL. The results were expressed as mg of caffeic acid equivalent (CAE) per 1 g of DE.

### 2.6. LC-ESI-MS/MS Analysis 

Phenolic acids and flavonoids contents were analyzed by liquid chromatography coupled with electrospray ionization tandem mass spectrometry (LC-ESI-MS/MS). For the chromatographic separation of the obtained extracts, an Agilent 1200 Series HPLC system (Agilent Technologies, Santa Clara, CA, USA) equipped with a binary gradient solvent pump, a degasser, an autosampler, and a column oven was used. The separation of analyzed compounds was performed with a Zorbax SB-C_18_ analytical column (2.1 × 100 mm × 1.8 µm; Agilent Technologies, Palo Alto, CA, USA). The column temperature was carried at 25 °C. Elution was conducted using solvent A (0.1% HCOOH in water) and solvent B (0.1% HCOOH in acetonitrile). The following gradient elution at a flow rate of 300 µL/min and 3-µL injection volume was used: 0–2 min—20% B; 3–4 min—25% B; 5–6 min—35% B; 5–6 min—35% B; 8–12 min—65% B; 14–16 min—80% B; and 20–28 min—20% B. 

MS detection was made in a 3200 QTRAP Mass spectrometer (AB Sciex, Framingham, MA, USA) with an electrospray ionization source (ESI) and a triple quadrupole-ion trap mass analyzer that was monitored by the Analyst 1.5 software. The ESI worked in the negative-ion mode, and the optimum values of the source parameters were: capillary temperature 450 °C, curtain gas 30 psi, nebulizer gas 50 psi, source voltage −4500 V for the phenolic acids and flavonoid glycosides, and capillary temperature 550 °C, curtain gas 20 psi, nebulizer gas 30 psi, and source voltage −4500 V for the analysis of flavonoid aglycones. Nitrogen was applied as collision and curtain gas. For each compound, the optimum conditions of multiple reaction mode (MRM) were established in the infusion mode. The analytes were identified by comparing the retention time and *m/z* values obtained by MS and MS^2^ with the mass spectra from corresponding references checked under the same conditions. The calibration curves from MRM mode were used for quantification of all samples. The identified compounds were quantified on the basis of their peak areas, and comparison with a calibration curve was obtained with the corresponding standards. Linearity ranges for the calibration curves were determined. The limits of detection (LOD) and quantification (LOQ) for all the samples were specified at signal-to-noise ratios of 3:1 and 10:1, respectively, using the injection of a series of dilute solutions with known concentrations [21]. Details of the conditions of LC-ESI-MS/MS analysis are shown in Table 1 and Table 2.

### 2.7. Cell Lines and Cell Culture

Human acute promyelocytic leukemia cell lines HL-60 (CCL 240^™^), HL-60/MX1 (CRL–2258^™^), and HL-60/MX2 (CRL–2257^™^), and acute lymphoblastic leukemia cell lines CEM/C1 (CRL-2265^™^) and CCRF/CEM (CCL–119^™^) were used in this research. Cell lines were from the American Type Culture Collection (ATCC^®^) 10801, University Boulevard Manassas, VA 20110, USA. Details regarding all the cell lines used in our study have been described previously by Kubrak et al. [22]. The cells were kept in RPMI 1640 medium (Biomed, Lublin, Poland) with 10% fetal bovine serum (FBS) (PAA Laboratories) for HL-60/MX1, HL-60/MX2, CEM/C1, and CCRF/CEM, and 20% FBS for HL-60 cell lines, streptomycin, and penicillin (100 U/mL PAA Laboratories), and 2.5 µg/mL amphotericin B (Gibco, Carlsbad, USA) at 37 °C in a humidified atmosphere of 5% CO_2_.

### 2.8. Analysis of Cell Viability

The cells of all the lines were put on 12-well plates (Sarstedt GesmbH, Wiener Neudorf, Austria) at an initial density of 1 × 10^6^ cells/mL. After incubation (24 h at 37 °C), the cell suspension was stimulated with investigated extracts at concentrations ranging from 1 to 1000 μg/mL for etheric extracts and from 1 to 5000 μg/mL for methanolic extracts. Then, 1 mL of cell suspension was centrifuged at 900 rpm for 5 min, the supernatant was discarded, and the cells were resuspended in 50 μL of PBS. Then, 10 μL of cell suspension was taken and mixed with 10 μL of 0.4% solution of Trypan blue reagent (Bio-Rad, Hercules, CA, USA). The samples were incubated for 5 min. Cell viability was measured using TC 10™ Automated Cell Counter (Bio-Rad). The experiment was done in triplicate. The IC_50_ (half-maximal inhibitory concentration, the inhibitor concentration when cell viability is 50%) values of both extracts were determined using MS Excel.

### 2.9. Antioxidant Activity Cell-Free Assays

All the assays were made using 96-well microplates (Nunclon, Nunc, Roskilde, Denmark) and were measured in an Elisa Reader Infinite Pro 200F (Tecan Group Ltd., Männedorf, Switzerland).

First, 2,2-diphenyl-1-picryl-hydrazyl (DPPH^•^) free radical scavenging activity of the extracts and the reference compound ascorbic acid was tested using a previously described method [23,24]. The DPPH^•^ absorbance decreasing induced by the samples was monitored at 517 nm. The second method used was 2,2′-azinobis[3-ethylbenzthiazoline]-6-sulfonic acid (ABTS^•+^) decolorization assay [25]. The absorbance was measured at 734 nm. Gallic acid was used as a positive control.

The hypochlorous acid (HOCl)-scavenging effect of the extracts and the reference compound ascorbic acid was studied by means of 5-thio-2-nitrobenzoic acid (TNB) as a reductant oxidized by HOCl into 5,5-dithiobis (2-nitrobenzoic acid) according to the method described earlier by Czerwińska et al. [26]. The absorbance was monitored at 412 nm. The scavenging of superoxide anion (O_2_^•^^−^) was determined by means of a xanthine–xanthine oxidase system with the NBT reduction assay according to the previously reported procedure of Kiss et al. [27]. Quercetin was used as a positive control. To estimate the samples influenced on the O_2_^•^^−^ production by direct interaction with xanthine oxidase, enzyme activity was made by monitoring the uric acid formation at 295 nm [28].

All the results were expressed as the IC_50_ values of the extracts on grounds on concentration–inhibition curves.

### 2.10. Statistical Analysis

All the results were expressed as means ± standard error of the mean (SEM) of three independent experiments. One-way ANOVA with Tukey’s post hoc test was used for the statistical analysis of significance of differences between means. *P* values below 0.05 were accepted as statistically significant. All the investigations were done by means of Statistica 10.0 (StatSoft Poland, Cracow, Poland).

## 3. Results and Discussion

### 3.1. Polyphenol Composition

There are only a few reports in the literature about the composition and biological activity of *Laserpitium* species [2,3,4,5,9,10,11,12,14,15,16,17,18]. To date, the polyphenol composition of fruits of *L. krapffii* have not been investigated. Therefore, the first stage of our study was to examine the total content of these compounds in the etheric and methanolic extracts of fruits of *L. krapffii*. The amounts of phenolic compounds in the tested extracts were proportional to the intensity of absorption measured spectrophotometrically. The analysis indicated that the methanolic extract contains much larger quantities of total phenolic, flavonoids, and phenolic acids content. The obtained results are shown in Table 3.

**Figure 1:**
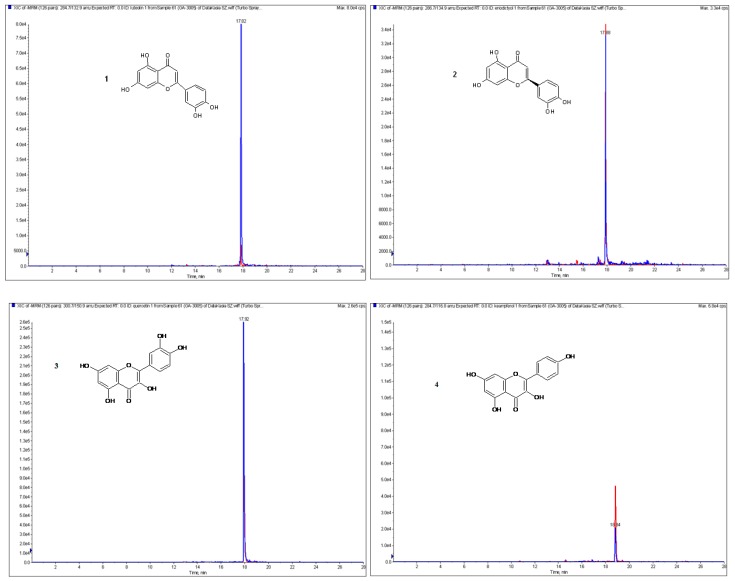
The chromatograms in multiple reaction mode (MRM) of flavonoid aglycones occuring in the methanolic extract of *L. krapffii* fruits: 1-luteolin; 2-eriodictyol; 3-quercetin; 4-kaempferol.

**Figure 2:**
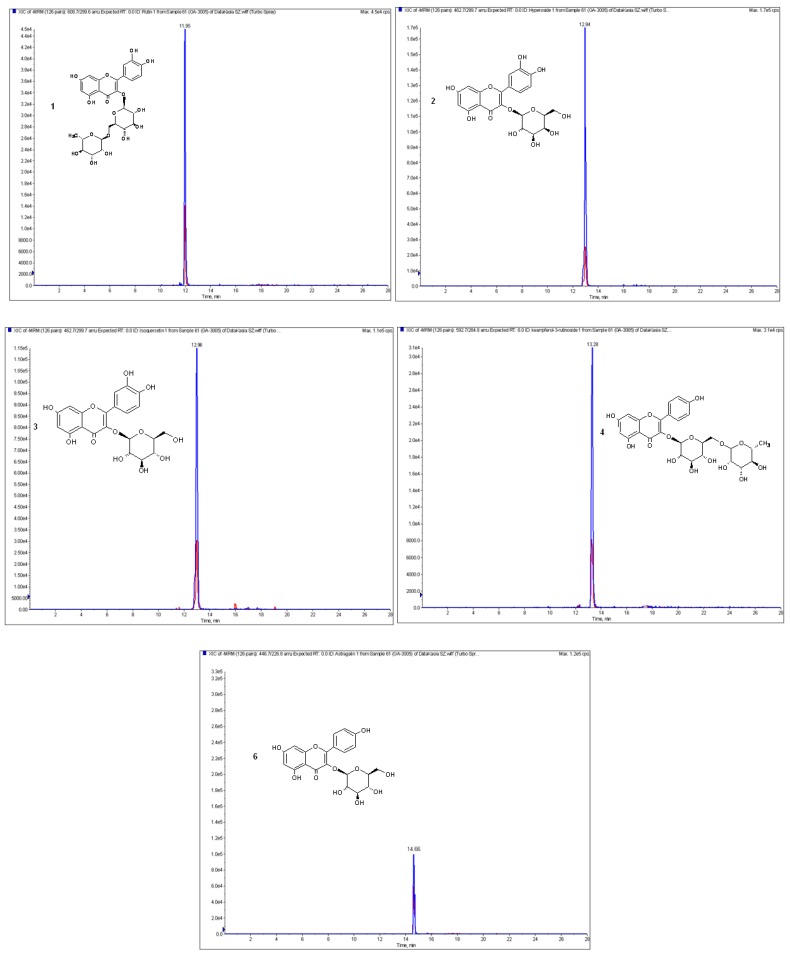
The chromatogram in MRM of flavonoid glycosides occurring in the methanolic extract of *L. krapffii* fruits: 1-rutin; 2-hyperoside; 3-isoquercetin; 4-kaempferol-3-*O*-rutinoside; 6-astragalin.

The next purpose of our study was the qualitative and quantitative analysis of phenolic acids and flavonoids in obtained extracts. The optimized LC-ESI-MS/MS procedure allowed the identification of 12 phenolic acids and nine flavonoids in the etheric and methanolic extracts from the fruits of *L. krapffii*. The chromatograms of the flavonoid aglycones and glycosides in methanolic extract are shown in Figure 1 and Figure 2, respectively. The amounts of all the compounds, which were quantified by a comparison of peak areas with the calibration curves of the corresponding references, are shown in Table 4. Both extracts did not differ significantly in the composition of polyphenols except for the absence of 3-hydroxybenzoic acid in the etheric extract. Gallic acid, eriodictyol, kaempferol, and nicotiflorin were identified in quantifiable amounts only in the methanolic extract.

Protocatechuic (LAM—195.93 ± 0.47 μg and LAEN—58.10 ± 1.16 μg per g of dry extract) and vanillic (LAM—34.13 ± 0.08 μg and LAEN—12.45 ± 0.21 μg/g DE) acids were the most abundant among the phenolic acids in both investigated extracts. In the methanolic extract, the large amount of 4-hydroxybenzoic (24.4 ± 0.03 μg/g DE), *p*-coumaric (24.3 ± 0.38 μg/g DE), and caffeic (20.55 ± 0.38 μg/g DE) acids was also observed.

Among the flavonoid aglycones, a large amount of quercetin (13.92 ± 0.03 μg/g of dry LAEN extract and 22.18 ± 0.09 μg/g of dry LAM extract) was found. The highest yields of flavonoid glycosides was obtained for the methanolic extract. Isoquercetin, nicotiflorin, and astragalin were presented in the greatest quantities in this extract.

To the best of our knowledge, *L. krapffii* was investigated for the first time in terms of phenolic acids and flavonoids quality and quantity. However, from the leaves of *L. latifolium*, astragalin, avicularin, isoquercitrin, quercitrin, rutin, chlorogenic and neochlorogenic acid, and other unidentified quercetin, isorhamnetin, and kaempferol derivatives were previously isolated [9].

Since plant phenolic acids and flavonoids have been noticed to possess a wide spectrum of biological activities including anti-inflammatory, antimutagenic, antioxidant, antitumor, and anticarcinogenic properties [29,30,31], the antioxidant and cytotoxic activity of the etheric and methanolic extracts obtained from the fruits of *L. krapffii* were also evaluated.

### 3.2. In Vitro Cytotoxicity Assay

The process of induction of neoplastic changes is associated with the appearance and accumulation of numerous mutations in the genetic material that caused the acquisition of unlimited division potential by phenotype cells. Such cells start to divide without limitations and lose their ability to die, creating a cancer. One way to eliminate cancer cells is to kill them by starting the process of apoptosis. The occurrence of multidrug resistance, inadequate efficacy, and numerous complications of current therapies motivate the search for new drugs. Plants are one of the basic sources of anti-cancer compounds [22,32].

**Figure 3:**
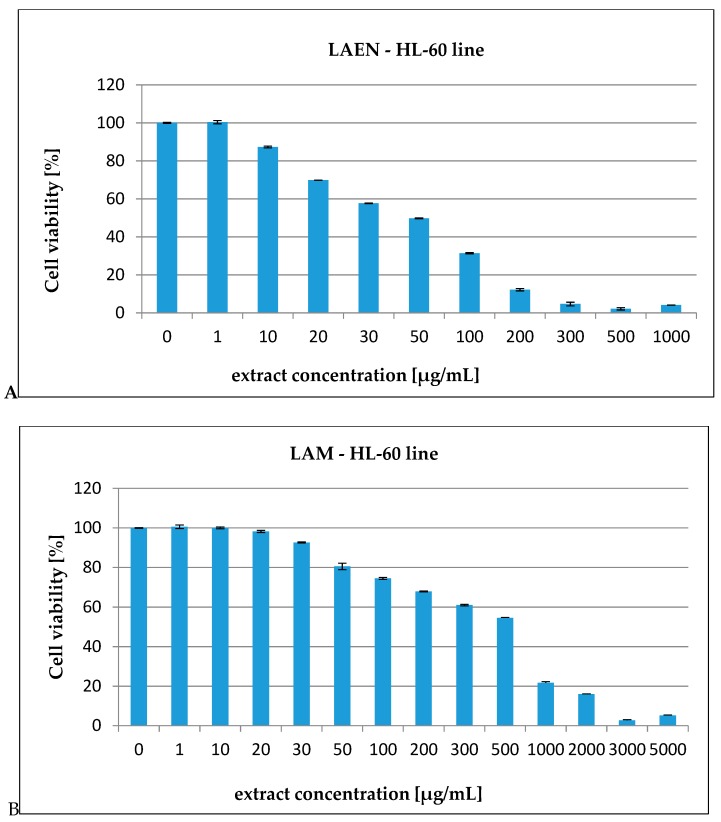
The valuation of the HL-60 cell line viability exposed to 24 h of increasing concentrations of: (**A)** — LAEN extract and (**B**) — LAM extract.

**Figure 4:**
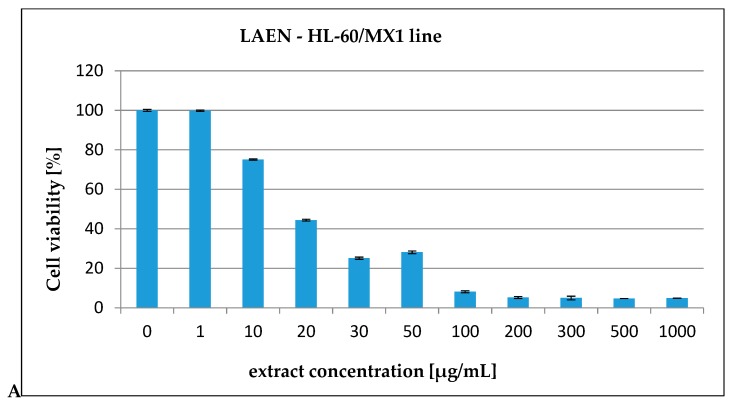

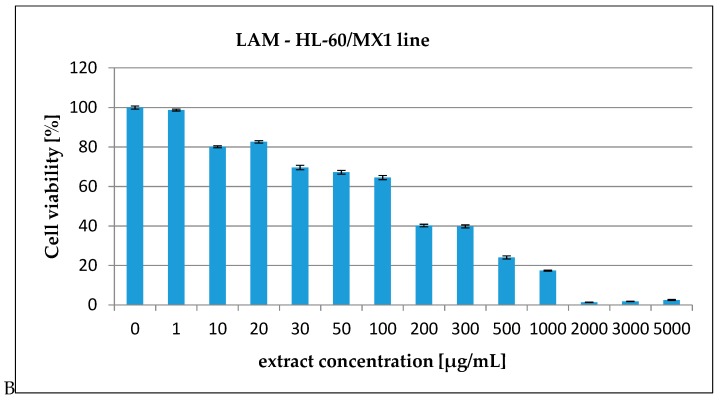
The valuation of the HL-60/MX1 cell line viability exposed to 24 h of increasing concentrations of: (**A**)—LAEN extract and (**B**)—LAM extract.

**Figure 5:**
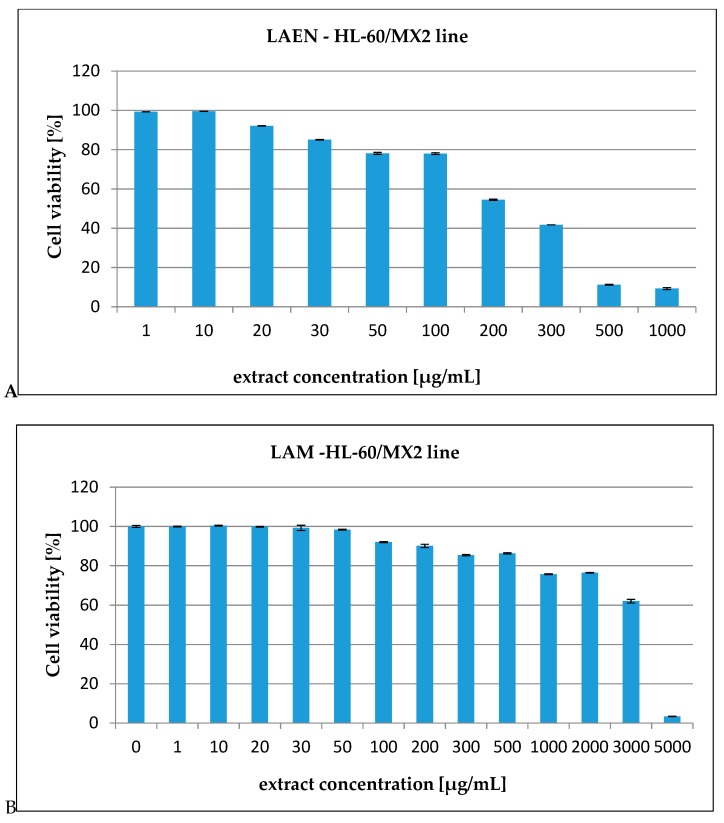
The valuation of the HL-60/MX2 cell line viability exposed to 24 h of increasing concentrations of: (**A**)—LAEN extract and (**B**)—LAM extract.

**Figure 6:**
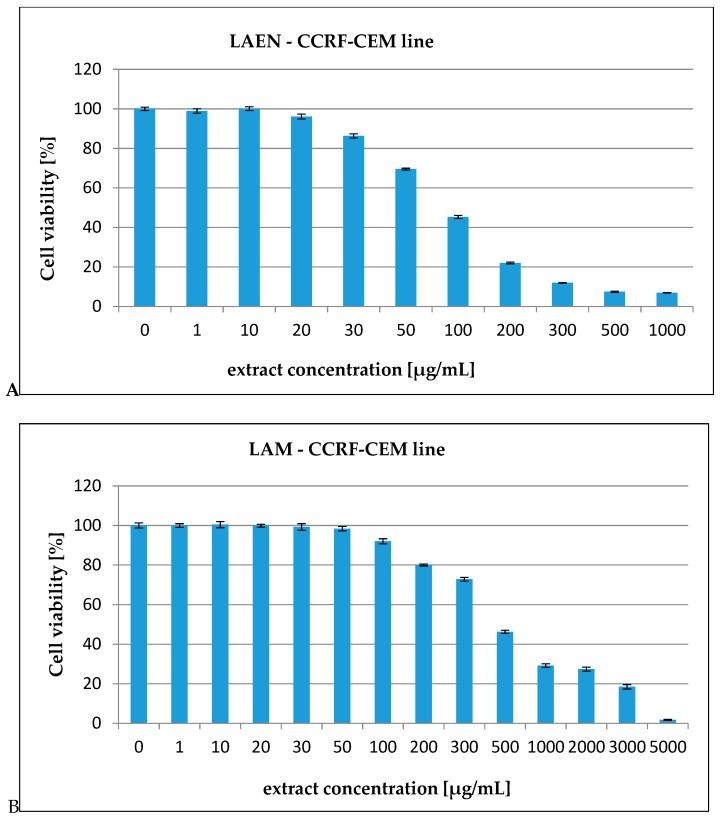
The valuation of the CCRF-CEM cell line viability exposed to 24 h of increasing concentrations of: (**A**)—LAEN extract and (**B**)—LAM extract.

**Figure 7:**
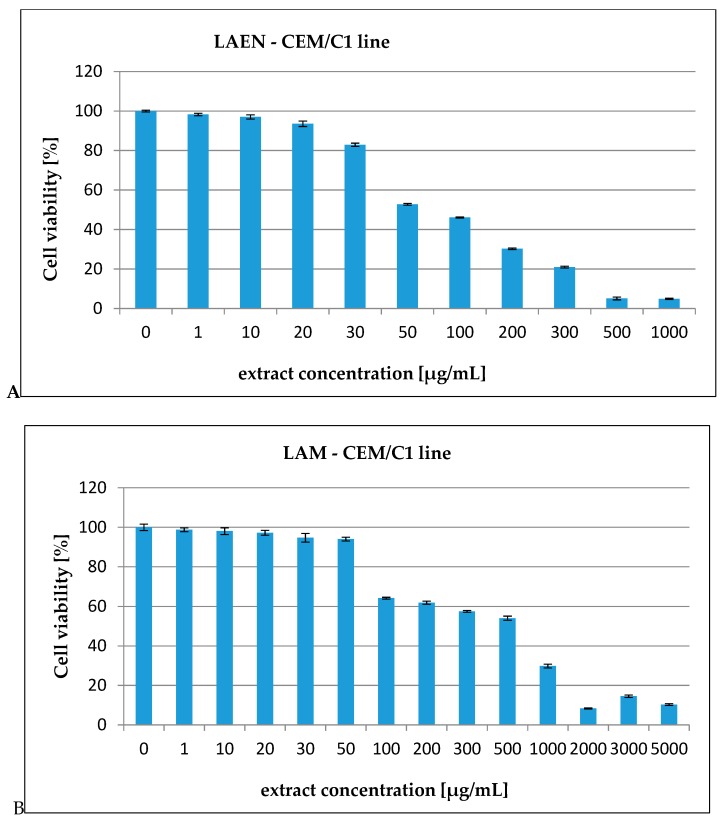
The valuation of the CEM/C1 cell line viability exposed to 24 h of increasing concentrations of: (**A**)—LAEN extract and (**B**)—LAM extract.

In our report, the effect of the etheric and methanolic extracts in increasing concentrations on five types of cancer cell lines, HL-60 (Figure 3), HL-60/MX1 (Figure 4), HL-60/MX2 (Figure 5), CEM/C1 (Figure 6), and CCRF/CEM (Figure 7) was investigated. The cytotoxicity was estimated using trypan blue vital staining. The cells of all the cancer lines exposed to the examined samples showed various cytotoxicity depending on the IC_50_ value.

Based on the obtained results, it was found that the analyzed extracts from the fruits of *L. krapffii* induce apoptosis of the cells of all the tested cell lines. The results, which are given in Table 5, showed that the etheric extract (LAEN) significantly inhibited HL-60/MX1 human leukemia cells, and was the most potent sample with an IC_50_ value of 31.08 μg/mL. Moreover, this extract showed a moderate cytotoxicity against HL-60 with IC_50_ = 72.19 μg/mL and against CEM/C1 with IC_50_ = 99.40 μg/mL. The etheric extract showed also relatively high cytotoxicity against CCRF/CEM (IC_50_ = 172.58 μg/mL).

The weak cytotoxic activity was found in the methanolic extract from the fruits of *L. krapffii*. The IC_50_ values for the LAM extract ranged from 205.72 μg/mL for the HL-60/MX1 line to 2848.31 μg/mL for the HL-60/MX2 line.

The presence of the large amounts of protocatechuic acid and quercetin may be responsible for the cytotoxic activity of the studied extracts. It has been reported that protocatechuic acid induces apoptosis in HL-60 human leukemia cells [33].

The published reports on the cytotoxic and antitumor activities of *Laserpitium* species are limited, and they focus on only a few species: *Laserpitium latifolium* L., *L. zernyi* Hayek, and *L.* *ochridanum* Micevski. The cytotoxic activities of chloroform extracts of dried roots and rhizomes of *L. zernyi* and *L.* *ochridanum* were tested against MCF 7/6 and MCF 7/AZ (human breast cancer) cell lines. The extracts exerted cytotoxic activities with the IC_50_ values ranging from 65.21 to 348.25 μg/mL. The obtained results showed that the extract of *L. ochridanum* was most potent in the MTT test with IC_50_ values of 65.21 μg/mL and 66.09 μg/mL in the MCF 7/AZ and MCF 7/6 cell lines, respectively [5].

Despite the use of different *Laserpitium* species and various cell lines as well as different research assays, the IC_50_ values obtained in our work and previous works are similar.

### 3.3. Antioxidant Activity

In our research, the antioxidant activity of etheric and methanolic extracts of the fruits of *L. krapffii* was determined for the first time. We used four colorimetric methods to determine the activity of both tested extracts against synthetic radicals DPPH^•^ and ABTS^•+^, xanthine/xanthine oxidase (generating O_2_^•^^−^), as well as against HOCl. The measurement of antioxidant activities was performed on a microplate scale in cell-free systems.

**Figure 8:**
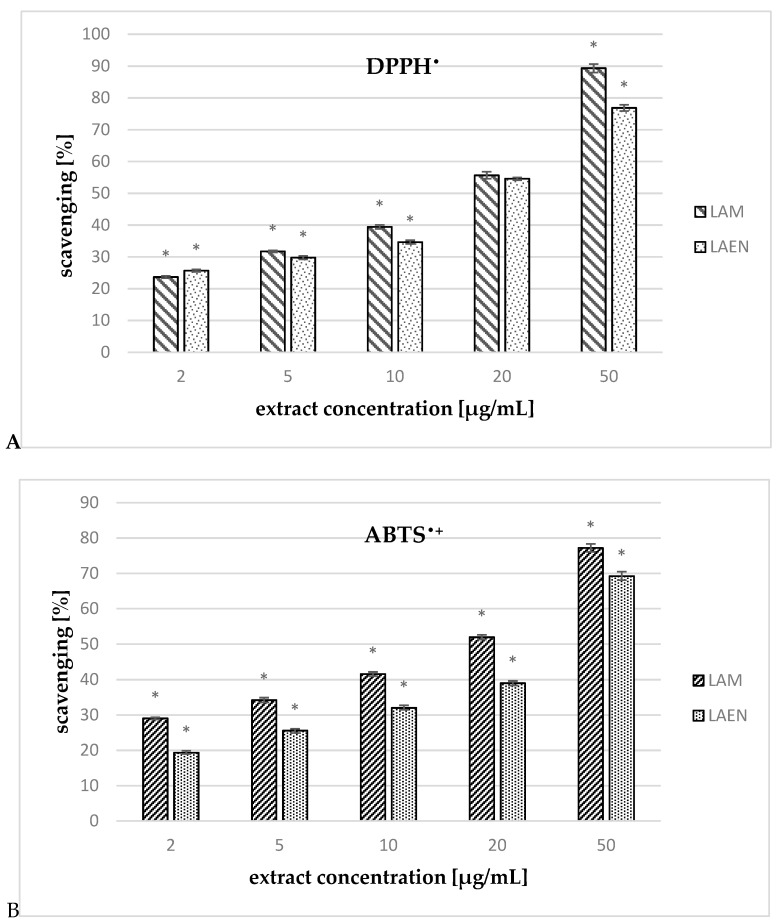
Antioxidant activity of the etheric (LAEN) and methanolic (LAM) extracts against: (**A**) 2,2-diphenyl-1-picrylhydrazyl radical (DPPH), and (**B**) 2,2′-azino-bis-(3-ethyl-benzthiazoline-6-sulfonic acid) (ABTS). Data are expressed as means ± SEM; obtained in three independent experiments, assayed in triplicate. Statistical significance of differences was calculated between both extracts; * *p* < 0.05.

The extracts examined in the study exhibited high scavenging ability in a concentration-dependent manner (Figure 8). For comparison, the radical scavenging activity of ascorbic acid was measured in the same conditions. The higher DPPH^•^ scavenging activity was demonstrated for the methanolic extract (LAM). The IC_50_ value for methanolic extract was almost one and half times lower than the for ascorbic acid (Table 6).

The percentage of ABTS^•+^ radicals reduction for extracts was measured at λ = 734 nm after 10 min of incubation (experimentally determined). In this time, both the analyzed extracts and the reference compound reached a constant antioxidant value. In comparison to gallic acid, which is a strong antioxidant compound [34], both tested extracts of *L. krapffii* showed weak antioxidant properties. The IC_50_ values for the methanolic (20.69 μg/mL) and etheric (30.45 μg/mL) extracts were much higher than that for gallic acid, which was used as a positive control (0.75 μg/mL).

**Figure 9:**
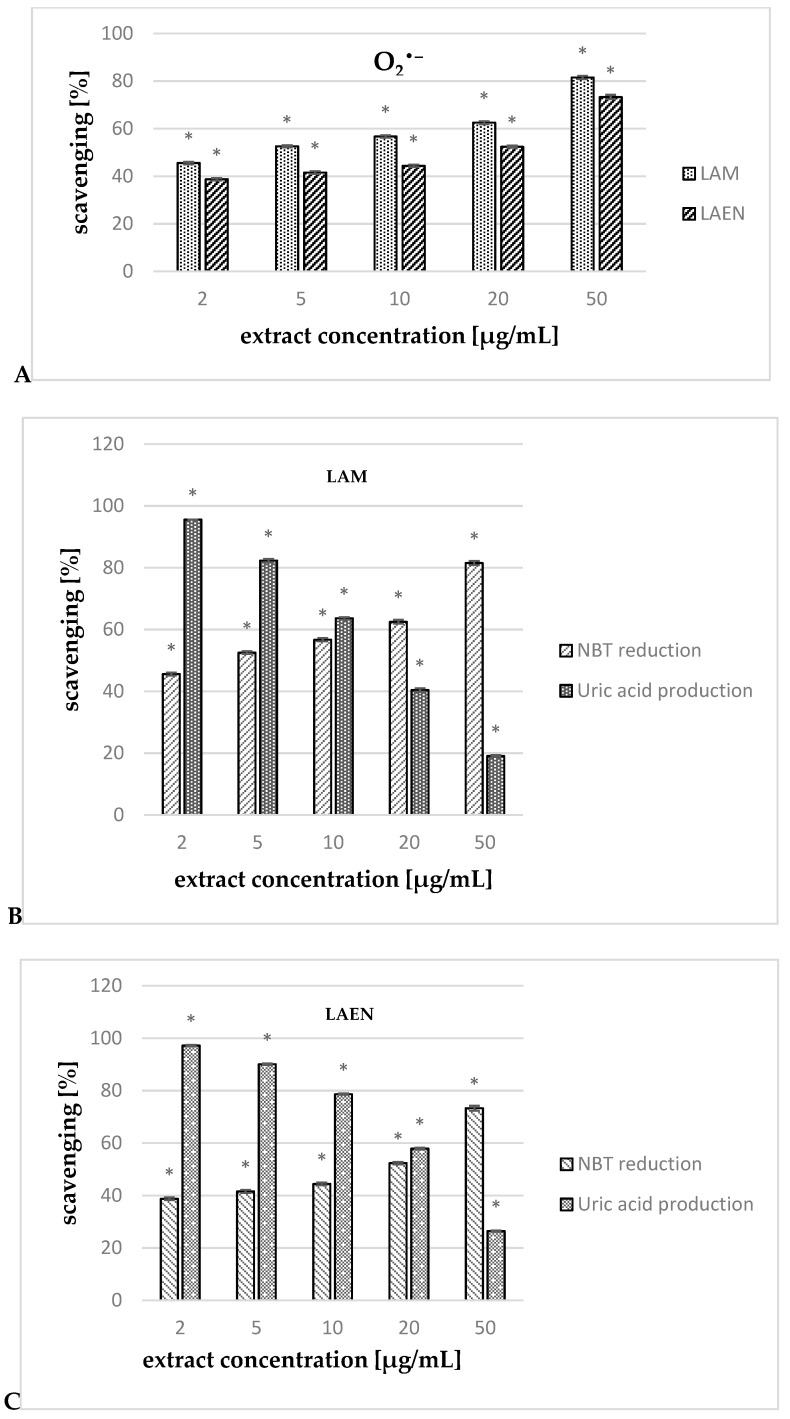
Scavenging effect of the etheric (LAEN) and methanolic (LAM), in the concentration range of 2 to 50 μg/mL, on O_2_^•^^−^ scavenging [%] in the xanthine/xanthine oxidase system (**A**). Effects of the LAM (**B**) and LAEN (**C**) on the xanthine/xanthine oxidase system [%]: nitrobluetetrazolium (NBT) reduction determines the scavenging of O_2_^•^^−^; uric acid production determines the oxidase inhibition. Statistical significance of differences was calculated between both extracts or between uric acid production and NBT reduction; * *p* < 0.05.

In the study, we also evaluated the ability of the etheric and methanolic extracts of fruits of *L. krapffii* to scavenge O_2_^•^^−^ generated by the xanthine/xanthine oxidase system. Both extracts scavenge O_2_^•^^−^ in a concentration-dependent manner (Figure 9A). At all the concentrations studied, LAM showed statistically (*p* < 0.05) stronger activity than the etheric extract did. The IC_50_ value for the methanolic extract (3.28 μg/mL) was almost five times lower than that for the etheric extract (17.31 μg/mL), and almost two times lower in comparison to the quercetin (5.72 μg/mL). Additionally, both extracts strongly inhibited uric acid production (for LAEN—97.23–26.41%, and for LAM—95.56–19.06%), confirming that their activity in a xanthine/xanthine oxidase system is related to the scavenging effect against O_2_^•^^−^ and the inhibition of the enzyme activity (Figure 9B,C).

**Figure 10:**
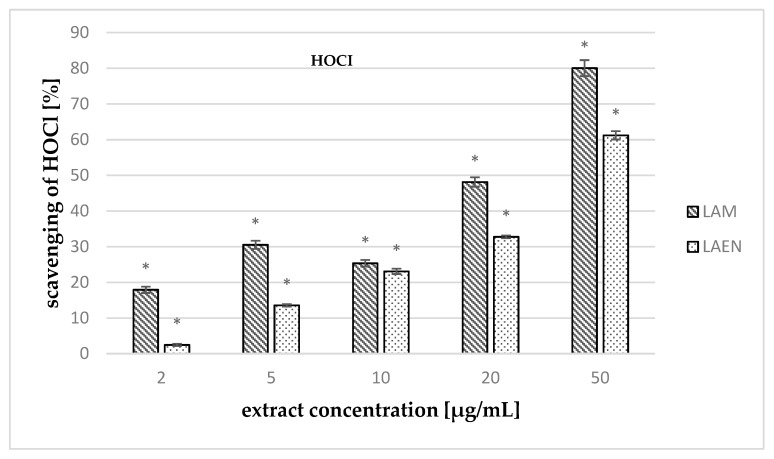
Scavenging effects of the etheric (LAEN) and methanolic (LAM) on hypochlorous acid (HOCl)-mediated oxidation of 5-thio-2-nitrobenzoic acid (TNB). Data are expressed as means ± SEM, obtained in at least three independent experiments, and assayed in triplicate. Statistical significance of differences was calculated between both extracts; * *p* < 0.05.

The measurement of activity against HOCl (Figure 10) showed that the extracts tested have a high scavenging ability of this radical. In the concentrations studied (2–50 μg/mL), the methanolic extract had stronger ability (17.91–80.04%) than the etheric one (2.45–61.23%), and the IC_50_ values were 25.09 μg/mL and 38.17 μg/mL, respectively. Moreover, the IC_50_ values for both extracts were lower than that for ascorbic acid, which was used as a reference compound (49.56 μg/mL).

The obtained lower IC_50_ values for the methanolic extract in all the antioxidant assays may be connected with higher phenolic compounds content, as it is well known that flavonoids and phenolic acids are the most popular natural components responsible for the antioxidant activity of plants.

## 4. Conclusions

In the literature, there are no reports concerning the biological activities of extracts of *Laserpitium krapffii*. In this study, it was found that both extracts of the fruits of *L. krapffii* have high cytotoxic and antioxidant potential, and can be a source of valuable compounds with protective effects against oxidative damage. The inhibiting influence of both extracts on ROS production may reduce oxidative modifications of biomolecules and tissue dysfunctions during chronic degenerative diseases.

## Figures and Tables

**Table 1:** Liquid chromatography–electrospray tandem mass spectroscopy (LC-ESI-MS/MS) analytical results of phenolic acids and flavonoids investigated in the extracts, which were confirmed by comparison with reference compounds.

Compound	Retention Time [min]	[M − H]^−^ [*m/z*]	Fragment Ions [*m/z*]	Collision Energy [eV]
Gallic acid	5.07	168.7	78.9 124.9	−36 −14
Protocatechuic acid	8.23	152.9	80.9 107.8	−26 −38
4-Hydroxybenzoic acid	11.27	136.8	92.9 107.9	−18 −18
Syringic acid	11.41	196.9	122.8 181.9	−24 −12
Gentisic acid	11.43	352.9	80.0 96.9	−110 −52
Vanilic acid	11.49	166.8	107.9 123.0	−18 −12
Caffeic acid	11.68	178.7	88.9 134.9	−46 −16
Rutin	11.99	608.7	299.6 270.9	−46 −60
3-Hydroxybenzoic acid	12.08	136.9	93.0 75.0	−16 −48
Hyperoside	12.80	462.7	299.7 254.7	−28 −42
Isoquercetin	13.00	462.7	299.7 270.7	−30 −44
Nicotiflorin	13.31	592.7	284.8 226.7	−38 −68
*p*-Coumaric acid	14.18	162.8	93.0 119.0	−44 −14
Astragalin	14.66	446.7	226.8 254.8	−54 −40
Ferulic acid	14.84	192.8	133.9 177.9	−16 −12
*o*-Coumaric acid	17.17	162.8	119.0 93.0	−14 −46
Luteolin	17.54	284.7	132.9 150.9	−38 −26
Eriodictyol	17.87	286.7	134.9 150.9	−32 −18
Quercetin	17.91	300.7	150.9 178.8	−26 −20
Salicylic acid	18.06	136.9	75.0 93.0	−48 −16
Kaempferol	18.56	284.7	116.8 93.0	−46 −52

**Table 2:** Analytical results of LC-MS/MS quantitative method for determination of flavonoids and phenolic acids. Limit of detection (LOD), limit of quantification (LOQ) and calibration curve parameters.

Compound	LOD [ng/mL]	LOQ [ng/mL]	*R* ^2^	Linearity Range [ng/mL]
Gallic acid	33.3	95.0	0.9987	167–3300
Protocatechuic acid	17.0	34.0	0.9997	34–3470
4-Hydroxybenzoic acid	17.4	34.7	0.9993	69.4–3470
Syringic acid	167.0	666.0	0.9993	666–11,100
Gentisic acid	1.7	3.3	0.9997	3.3–330
Vanillic acid	100.0	250.0	0.9997	330–33,000
Caffeic acid	60.0	160.0	0.9990	175–3500
Rutin	120.0	300.0	0.9985	2000–25,000
3-Hydroxybenzoic acid	33.3	334.0	0.9994	334–6670
Hyperoside	150.0	200.0	0.9987	1000–25,000
Isoquercetin	150.0	300.0	0.9987	2000–25,000
Nicotiflorin	60.0	120.0	0.9991	120–50,000
*p*-Coumaric acid	7.3	18.1	0.9996	18.1–1820
Astragalin	100.0	200.0	0.9978	1200–24,000
Ferulic acid	17.4	34.7	0.9994	69.4–11,600
*o*-Coumaric acid	7.3	18.1	0.9996	18.1–1820
Luteolin	25.0	40.0	0.9989	40–4000
Eriodictyol	10.0	15.0	0.9982	15–5000
Quercetin	5.0	10.0	0.9980	20–3000
Salicylic acid	3.3	16.5	0.9989	16.5–1650
Kaempferol	20.0	33.0	0.9989	33–20,000

**Table 3:** The total phenolic (TPC) and total flavonoid (TFC) content in the etheric (LAEN) and methanolic (LAM) extracts from *L. krapffii* fruits. The results are expressed as mg of gallic acid equivalent (GAE) and quercetin equivalent (QE) per 1 g of dry extract (DE). Values are presented in mean ± SEM, *n* = 3.

Extract	TPC [mg GAE/g DE]	TFC [mg QE/g DE]	TPAC [mg CAE/g DE]
LAEN	5.53 ± 0.58	0.41 ± 0.17	1.19 ± 0.06
LAM	14.53 ± 0.60	1.22 ± 0.38	7.98 ± 0.07

**Table 4:** Content of phenolic acids and flavonoid aglycones and glycosides in the etheric (LAEN) and methanolic (LAM) extracts from *L. krapffii* fruits. Values are presented in means ± SEM, *n* = 3. nd—not detected; BQL—peak detected, concentration lower than the LOQ but higher than the LOD.

Compound	LAEN	LAM
	Phenolic Acid/Flavonoid (μg per g of Dry Extract)	
Gallic acid	BQL	1.19 ± 0.05
Protocatechuic acid	58.10 ± 0.67	195.93 ± 0.27
4-Hydroxybenzoic acid	5.76 ± 0.17	24.4 ± 0.02
Syringic acid	1.60 ± 0.05	9.02 ± 0.02
Gentisic acid	1.53 ± 0.13	8.33 ± 0.14
Vanillic acid	12.45 ± 0.12	34.13 ± 0.05
Caffeic acid	5.34 ± 0.08	20.55 ± 0.22
Rutin	3.17 ± 0.02	11.05 ± 0.42
3-Hydroxybenzoic acid	nd	0.73 ± 0.08
Hyperoside	1.89 ± 0.00	5.12 ± 0.08
Isoquercetin	3.05 ± 0.06	22.53 ± 0.01
Nicotiflorin	BQL	15.36 ± 0.02
*p*-Coumaric acid	6.65 ± 0.18	24.3 ± 0.22
Astragalin	0.74 ± 0.03	15.51 ± 0.01
Ferulic acid	0.75 ± 0.05	2.91 ± 0.08
*o*-Coumaric acid	2.56 ± 0.05	9.58 ± 0.32
Luteolin	0.16 ± 0.00	0.86 ± 0.08
Eriodictyol	BQL	0.17 ± 0.02
Quercetin	13.92 ± 0.01	22.18 ±0.05
Salicylic acid	3.83 ± 0.24	13.35 ± 0.25
Kaempferol	BQL	0.04 ± 0.00

**Table 5:** The extrapolated IC_50_ values for the HL-60, HL-60/MX1, HL-60/MX2, CEM/C1, and CCRF/CEM line cells.

-	IC_50_ [µg/mL]	
	LAEN	LAM
HL-60	72.19	757.77
HL-60/MX1	31.08	205.72
HL-60/MX2	248.43	2848.31
CEM/C1	99.40	513.03
CCRF/CEM	172.58	671.90

**Table 6:** The IC_50_ values established in antioxidant assays. Data are expressed as mean ± SEM, *n* = 3. AA—ascorbic acid, GA—gallic acid, Q quercetin.

Assay	Antioxidant Activity [IC_50_ in μg/mL of Extract]				
	LAEN	LAM	AA	GA	Q
DPPH^•^	22.70 ± 0.23	18.93 ± 0.34	25.01 ± 0.14	-	-
ABTS^•+^	30.45 ± 0.47	20.69 ± 0.43	-	0.75 ± 0.01	-
O_2_^•−^	18.35 ± 0.16	3.28 ± 0.12	-	-	5.72 ± 0.02
HOCl	38.17 ± 0.02	25.09 ± 0.10	49.56 ± 1.35	-	-
